# Restoring Theoretically Optimal Lumbar Lordosis Deduced from Pelvic Incidence and Thoracic Kyphosis has Advantages to Decrease the Risk of Postoperative Mechanical Complications in Adult Spinal Deformity

**DOI:** 10.3389/fsurg.2022.860564

**Published:** 2022-04-11

**Authors:** Jingyu Wang, Qianshi Zhang, Fubing Liu, Hui Yuan, Yi Zhang, Xiaobin Wang, Jing Li

**Affiliations:** Department of Spine Surgery, Spinal Deformity Center, The Second Xiangya Hospital of Central South University, Changsha, China

**Keywords:** lumbar lordosis, pelvic incidence, thoracic kyphosis, adult spinal deformity, mechanical complications

## Abstract

**Objective:**

To establish a regression formula for LL based on individual PI and TK in asymptomatic population aged over 50 years and evaluate its predictive power for the occurrence of postoperative mechanical complications in patients with adult spinal deformity (ASD).

**Methods:**

A total of 178 asymptomatic adults were recruited for the study. The association between LL and PI, LL and TK, was investigated to establish a predictive formula for ideal LL based on PI and TK. Additionally, 93 ASD patients undergoing posterior correction surgery were retrospectively analyzed. The absolute value of the gap between postoperative actual LL and theoretical LL was defined as ΔLL. Patients were classified into two groups depending on the presence or absence of mechanical complications. The demographic and radiological data of patients were compared between the two groups.

**Results:**

A significant association was found between LL and PI (*r *= 0.599, *P < *0.001), LL and TK (*r *= 0.523, *P < *0.001). A novel formula was developed as follows: LL = 0.7*PI + 0.4*TK + 1 (*R*^2 ^= 0.524). In the validation cohort, 29 patients developed mechanical complications. Postoperative ΔLL (12.5 ± 7.6° vs. 7.0 ± 5.4°, *P *= 0.001) significantly increased the incidence of mechanical complications. The most appropriate threshold of ΔLL for predicting mechanical complications was 9.8°. For patients whose ΔLL were *<*9.8° and >9.8°, the incidence of mechanical complications was 19.4% and 54.8%, respectively.

**Conclusion:**

Ideal lumbar lordosis should be matched for PI and TK. The developed prediction formula for LL based on PI and TK in asymptomatic adults may help surgeons to understand the mechanisms of lumbar alignment generation and predict occurrence of mechanical complications after ASD surgery.

## Introduction

The prevalence of adult spinal deformity (ASD) in the general population has been reported to range from 1.4 to 32%, reaching 68% in older patients (1). Clinically, ASD presents with three-dimensional deformity of the spine and poor quality of life in patients. Surgical treatment can improve global balance and clinical symptoms of ASD patients. However, surgery may increase the risk of mechanical complications such as proximal junctional kyphosis (PJK), proximal junctional failure (PJF), and instrumentation failure by 20%–50% in ASD patients (2). Patients with serious complications require repeat surgery, which adds financial pressure on their family and society (3). Therefore, it is crucial to predict and develop strategies to avoid these complications.

Mechanical complications of ASD surgery are heterogenous and multifactorial (4). Majority of studies have reported that restoration of “normal sagittal alignment” should be the critical goal of ASD surgery as this can significantly decrease the incidence of postoperative mechanical complications (5–7). Previously, a series of algorithms were constructed to guide the reconstruction of lumbar lordosis (LL). In a study by Sebaaly et al. (8), it was found that the formulae for predicting LL solely based on pelvic incidence (PI) or thoracic kyphosis (TK) did not accurately predict the occurrence of mechanical complications; however, they reported that the global sagittal alignment satisfying the formula PI + LL + TK* < *45° could predict PJK with high sensitivity.

Accumulating evidence shows that LL is determined by pelvic incidence (PI), and is correlated with thoracic kyphosis (TK) (9–15). To date, several predictive models for ideal LL based on individual PI in asymptomatic adults of different races have been proposed (11, 15, 16). Nevertheless, few studies have tested the prediction performance of combined PI and TK in LL. Moreover, the studies were carried out using cohorts with young and middle-aged population. The parameters for sagittal alignment restoration may vary across ages (17). Therefore, a prediction formula for LL should be developed for elderly population.

The primary aim of this study was to establish a prediction formula for LL based on individual PI and TK in asymptomatic population aged over 50 years and evaluate its ability to predict occurrence of postoperative mechanical complications in ASD patients.

## Materials and Methods

### Patient Population

In the current study, 178 asymptomatic patients aged over 50 years were prospectively recruited at our institution between November, 2020 and August, 2021. The inclusion criteria of patients were as follows: (1) no spinal pathology or deformity; (2) no history of pelvic, hip, or lower limbs disease; (3) no history of spinal surgery; (4) no neurological or neuromuscular disorder.

To verify ability of the established formula to predict occurrence of mechanical complications, 93 ASD patients undergoing posterior pedicle screw correction between January 2010 and January 2019 were retrospectively analyzed. The inclusion criteria were: (1) age >50 years; (2) UIV at T9 to T11; (3) lower instrumented vertebrae (LIV) at sacrum/iliac; (4) complete full-spine radiographs (preoperative, immediate postoperative and at the last follow-up); (5) follow-up time ≥ two years. Exclusion criteria were: (1) incomplete medical records; (2) scoliosis secondary to infection, tumor, trauma, or neuromuscular scoliosis.

This study was approved by our institutional ethics committee, and informed consent was obtained from each participant.

### Data Collection

Demographic data, including gender, age, and body mass index (BMI) were collected from all participants.

Full-spine X-rays were obtained from each participant in erect standing posture with a clavicle position (the elbows fully bent with hands placing into the supraclavicular fossae) or 90°position (the arms and elbows straight out with hands gently grasping a bar) (18).

Using Surgimap (Spine Software, version 2.3.2.1, New York, NY), the following radiographical parameters were measured: (1) PI, angle subtended by a perpendicular line from the midpoint of the S1 endplate and a line connecting this point to the center of femoral heads; (2) pelvic tilt (PT), angle between the plumb line and the line connecting the femoral head axis and center of the sacral plate; (3) sacral slope (SS), angle between the horizontal line and superior endplate of S1; (4) LL, Cobb angle bound by the superior endplate of L1 and S1; (5) TK, Cobb angle formed between the superior endplate of T5 and the inferior of T12; (6) T1 pelvic angle (TPA), the angle formed by two lines from the femoral head axis to the midpoint of the S1 endplate and the center of T1.

All radiographical parameters were measured by two spinal surgeons, and mean values were used for data analysis. Intra- and inter-observer reproducibility was assessed in 50 randomly selected patients based on intraclass correlation coefficients (ICC). The intra-observer ICCs for PI, PT, SS, LL, TK, TPA were 0.902, 0.925, 0.932, 0.961, 0.903, and 0.914, whereas inter-observer ICCs for the aforementioned parameters were 0.928, 0.931, 0.910, 0.935, 0.892, and 0.937, respectively. Both intra- and inter-observer reliability were satisfactory according to the Shrout and Fleiss criteria (19).

### Development and Validation of the Predictive Model

The association of LL with PI and TK was investigated in the derivation cohort to establish the prediction formula for LL based on individual PI and TK in asymptomatic population.

The presence and type of mechanical complications were recorded (PJK, PJF, instrumentation failure not related to PJF such as rod breakage and screw pull-out or breakage) in the validation cohort. PJK was defined as postoperative proximal junctional angle (PJA) ≥ 10°, and at least 10° greater than its corresponding preoperative value (20). PJF was defined as implant-bone interface failure at the UIV, and fracture of the UIV or UIV + 1 (21). Patients were classified into two groups based on the presence or absence of mechanical complications.

Then, the ideal LL of patients in the validation cohort was determined using postoperative PI and TK according to the established formula, and the absolute value of the gap between ideal LL and actual LL was defined as ΔLL.

### Statistical Analysis

All statistical analyses were performed using SPSS 23.0 (SPSS Inc., Chicago, Illinois, USA). Quantitative data were expressed as mean ± standard deviation, and qualitative data were expressed as frequencies or percentages. The correlations between LL and other parameters was determined by the Pearson correlation analysis. Moreover, stepwise multiple linear regression was performed to predict LL (as the dependent variables) based on PI and TK (as the independent variables). Differences between groups were compared with an independent sample t-test and χ^2^ test. Receiver operating characteristics (ROC) curve was plotted to determine the best cut-off value of ΔLL as a predictor of mechanical complications. A *p*-value of <0.05 was considered statistically significant.

## Results

**Figure 1:**
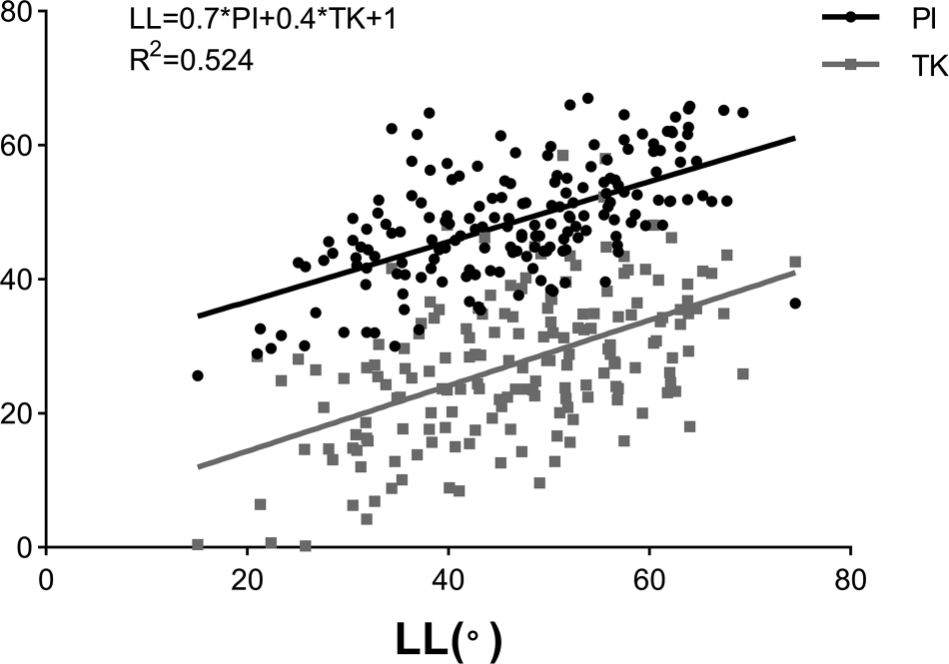
Linear correlations of lumbar lordosis (LL) with pelvic incidence (PI) and thoracic kyphosis (TK).

Among 178 patients in the derivation cohort, 87 were males and 91 were females. The mean age and BMI were 61.3 ± 5.8 years and 23.8 ± 2.6 kg/m^2^, respectively. The average values of radiographical parameters were 48.6 ± 8.7° for PI, 27.4 ± 10.9° for TK, and −46.7 ± 11.6° for LL (Table 1). Significant correlation was found between LL and PI (*r *= 0.599, *P < *0.001), LL and TK (*r *= 0.523, *P <* 0.001) (Table 2). Additionally, a weak correlation between PI and TK (*r *= 0.201, *P *= 0.007, TK = 0.3*PI + 14.8, *R*^2 ^= 0.043) was found. Results of multivariate analysis revealed that PI and TK were two significant predictors in the LL model (Table 3). A novel formula was developed as follows: LL = 0.7*PI + 0.4*TK + 1(*R*^2 ^= 0.524) (Figure 1).

**Table 1:** Demographic and spinopelvic parameters of the asymptomatic subjects.

Variables	Mean	Minimum	Maximum	Standard deviation
Age (years)	61.3	52.0	83.0	5.8
Gender (male/female)	87/91			
BMI (kg/m^2^)	23.8	17.0	30.8	2.6
LL (°)	−46.7	−15.1	−74.5	11.6
TK (°)	27.4	0.2	58.5	10.9
PI (°)	48.6	25.6	67.0	8.7

*BMI, body mass index; LL, lumbar lordosis; TK, thoracic kyphosis; PI, pelvic incidence.*

**Table 2:** Correlation between lumbar lordosis and the variables.

Variables	Correlation Coefficient (r)	*P* value
PI (°)	0.599	**<*0***.***001***
TK (°)	0.523	**<*0***.***001***

*PI, pelvic incidence; TK, thoracic kyphosis.* *The bold P-values indicate statistically significant.*

**Table 3:** Multiple linear regressions.

Dependent variables	Independent variables	B	Standard error	*t*	*P*	*R^2^*
LL (°)	Constant	1.251	3.540	0.353	0.724	0.524
	PI (°)	0.683	0.071	9.601	**<*0***.***001***	
	TK (°)	0.445	0.057	7.807	**<*0***.***001***	

*LL, lumbar lordosis; PI, pelvic incidence; TK, thoracic kyphosis.* *The bold P-values indicate statistically significant.*

In total, 93 ASD patients were included in validation cohort and the mean follow-up period was 2.71 ± 0.60 years. Patients in the derivation cohort and validation cohort were matched in terms of age, gender, and BMI (Table 4). Mechanical complications occurred in 29 patients (PJK (*n* = 18), PJF (*n* = 6), screw pull-out or breakage (*n* = 4), rod breakage (*n* = 1)).

**Table 4:** Demographic characteristics of the 2 cohorts.

Variables	Derivation Cohort	Validation Cohort	*P* value
Age (years)	61.3 ± 5.8	62.4 ± 4.5	0.085
Gender (male/female)	87/91	41/52	0.453
BMI (kg/m^2^)	23.8 ± 2.6	24.0 ± 2.3	0.482

*BMI, body mass index.*

Univariate analysis showed that patients with mechanical complications were significantly (*P *= 0.017) older than those without complications (64.0 ± 5.0 vs. 61.6 ± 4.1 years). Analysis of the radiographic parameters showed that a higher postoperative ΔLL (12.5 ± 7.6° vs. 7.0 ± 5.4°, *P *= 0.001) was significantly associated with increased incidence of mechanical complications. There were no significant differences in other parameters between patients with and without mechanical complications (Table 5).

**Table 5:** A comparison of the demographics and spinopelvic parameters between patients with and without mechanical complications.

Variables	Mechanical complications (*n* = 29)	No mechanical complications (*n* = 64)	*P* value
Demographics			
Age (years)	64.0 ± 5.0	61.6 ± 4.1	***0***.***017***
Gender (male/female)	16/13	25/39	0.147
BMI (kg/m^2^)	24.1 ± 2.5	24.0 ± 2.2	0.835
Follow-up (years)	2.8 ± 0.6	2.7 ± 0.6	0.616
Preoperative parameters (°)			
TPA	28.9 ± 14.2	25.4 ± 11.1	0.250
TK	17.9 ± 12.0	16.2 ± 11.7	0.514
LL	−21.6 ± 19.1	−27.0 ± 20.2	0.232
PI	51.4 ± 11.4	50.1 ± 12.1	0.636
PT	28.0 ± 10.9	24.5 ± 9.0	0.111
SS	23.4 ± 10.8	25.6 ± 9.9	0.337
PI-LL	29.8 ± 21.7	23.2 ± 18.3	0.132
Postoperative parameters (°)			
TPA	16.2 ± 11.6	13.8 ± 8.6	0.332
TK	24.5 ± 9.3	21.9 ± 6.9	0.186
LL	−42.3 ± 15.6	−43.3 ± 11.1	0.758
Ideal LL	−46.9 ± 8.0	−44.7 ± 8.9	0.258
ΔLL	12.5 ± 7.6	7.0 ± 5.4	***0***.***001***
PI	51.6 ± 10.9	49.9 ± 11.6	0.516
PT	21.5 ± 12.6	17.9 ± 9.7	0.173
SS	30.1 ± 12.4	32.0 ± 9.9	0.411
PI-LL	9.3 ± 16.5	6.6 ± 10.4	0.431

*BMI, body mass index; TPA, T1 pelvic angle; TK, thoracic kyphosis; LL, lumbar lordosis; PI, pelvic incidence; PT, pelvic tilt; SS, sacral slope.*

Ideal LL, 0.7*postoperative PI *+* 0.4* postoperative TK *+* *1; ΔLL, the absolute value of the gap between actual LL and ideal LL. The bold P-values indicate statistically significant.*

**Figure 2:**
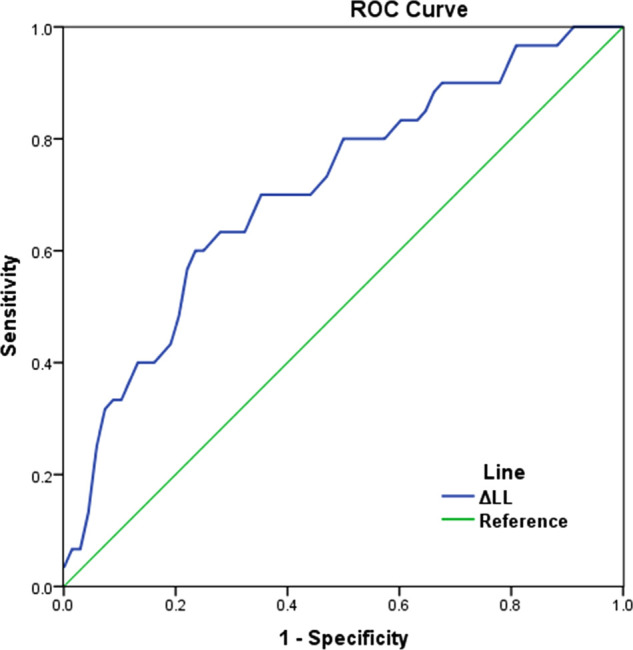
ROC curve showing the performance of ΔLL in predicting mechanical complications (the area under curve (AUC) was 0.721, the optimal cut-off value of ΔLL was 9.8°). ΔLL: the absolute value of the gap between actual LL and ideal LL.

Analysis of the ROC curve (Figure 2) showed that the optimal threshold of ΔLL as predictor for mechanical complications was 9.8°. Among patients whose ΔLL were *<*9.8° and >9.8°, the incidence of mechanical complications was 19.4% (12/62) and 54.8% (17/31), respectively.

## Discussion

Restoration of reasonable sagittal alignment has been demonstrated to remarkably decrease the incidence of mechanical complications (22). The target parameter PI-10° < LL < PI + 10° is commonly used in the reconstruction of sagittal alignment, however, it yields inconsistent results when applied in the prediction of mechanical complications (21, 22). In contrast, LL-TK combined with PI-LL is considered to be more effective in predicting sagittal balance in elderly population (13). Sagittal alignment may be regarded as an open linear chain connecting the pelvis with the head where the morphology of each successive anatomic segment are closely related and affect the adjacent segment (23). Therefore, it may not be sufficient to consider only the matching relationship between LL and pelvic morphology when evaluating whether LL is reasonable. Although several formulae for predicting LL based on PI and TK have been proposed, such as maxLL = −2.72–1.1PI + 1.1PT-0.31maxTK (24) and LL < 45°-TK-PI (9), these formulae either incorporate parameters that change with the position of the spine and pelvis, or simply a rough algebraic sum of the parameters, which may not accurately predict the ideal LL.

A total of 178 asymptomatic Chinese adults over 50 years old were recruited to develop a more accurate prediction formula of LL. In line with the findings of previous studies (11, 12, 14), significant correlations between LL and PI (*r *= 0.599, *P < *0.001), LL and TK (*r *= 0.523, *P < *0.001) were obtained. Theoretically, the pelvis and thoracic spine can be considered to be independently growing structures since they have unique morphologies due to pelvic cavity and rib cage development rather than for an erect posture (25, 26); previous studies also suggested that TK was not correlated with PI (23, 24, 27). Herein, a weak correlation between PI and TK (*r *= 0.201, *P *= 0.007, TK = 0.3*PI + 14.8, *R*^2 ^= 0.043) was found, suggesting that a more or less indirect interaction might occur among PI and TK due to a bridging function of the lumbar spine; however, the effect (*R*^2 ^= 0.043) is not significant in general.

**Figure 3:**
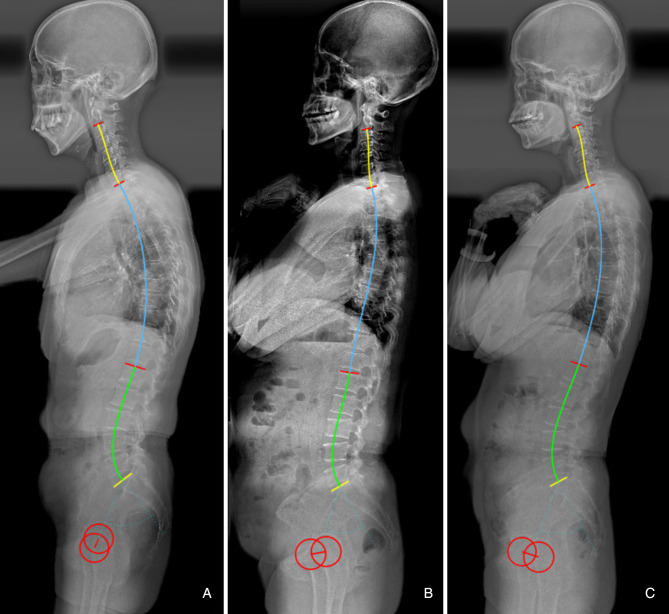
(**A**) Male, 60 years old, with a large pelvic incidence (PI) (60.1°) and thoracic kyphosis (TK) (33.9°) resulting a hyperlordosis (−55.5°). (**B**) Female, 62 years old, with a moderate PI (48.3°) and flat TK (8.9°), resulting a flat LL (−38.5°). (**C**) Female, 53 years old, with a similar PI (49.1°) to b, but a larger TK (27.9°), resulting a larger LL (−46.0°).

Multivariate regression analysis showed that LL = 0.7*PI + 0.4*TK + 1 (*R*^2^ = 0.524), which can intuitively reflect the matching relationship of LL with PI and TK. In other words, increased PI and TK should be accompanied by a hyperextension of lordosis to accommodate the aforementioned structures, and vice versa (Figures 3A,B). Besides, even individuals with similar PI will present different magnitudes of LL due to variance in TK (Figures 3B,C). Therefore, varying lumbar alignment can be attributed to the diversity of pelvic and thoracic morphology. Of note, the prediction efficiency of the formula (*R*^2^) was 0.524, indicating that 52.4% of the total difference observed in LL can be explained by PI and TK. Thus, the developed formulae outperformed the formulae containing a single variable, such as LL = 0.548*TK + 25.610 (*R*^2 ^= 0.276) (12) and LL = 0.588*PI + 21.0 (*R*^2 ^= 0.267) (14). Moreover, the variables in the formula are all morphological parameters which are independent of pelvic retroversion. Since the inflection point is variable, especially in pathological conditions, we measured LL and TK based on traditional anatomical landmarks, which seems to be simpler and convenient for application.

Sagittal alignment overcorrection and undercorrection have both been considered to be a main causes of mechanical complications (22). Therefore, patients should be stabilized in a position that does not require or require minimum compensation after corrective surgery. We speculated that postoperative global sagittal alignment which did not satisfy the formula might increase the occurrence of mechanical complications. To investigate the predictive ability of the formula for mechanical complications, a cohort of patients who underwent ASD surgery was analyzed. The 2 cohorts were well matched in terms of demographic information. Besides, to keep the groups as homogeneous as possible, we only included patients with long-segment fusion from the lower thoracic spine to the sacrum/iliac. The incidence of mechanical complications was 31.2%, and there was higher risk of complications in older patients, which is in line with findings from majority of previous studies (6, 22). Moreover, we analyzed the absolute value of the gap between actual LL and the theoretical value derived from postoperative PI and TK. The results showed that a mismatch between postoperative LL and the ideal LL, which was significantly associated with occurrence of mechanical complications (54.8% vs. 19.4%), illustrating that the formula can effectively predict postoperative mechanical complications.

**Figure 4:**
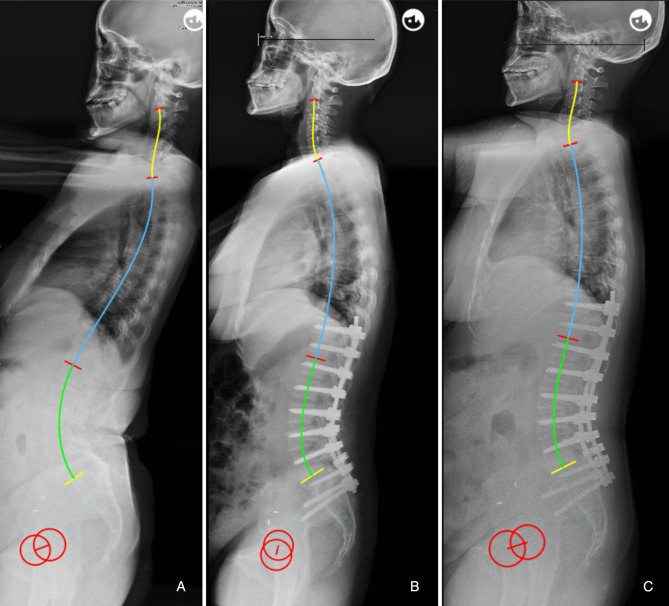
60-year-old female (**A**) preoperative sagittal standing full spine X-rays, pelvic incidence (PI) = 58.5°, thoracic kyphosis (TK) = 8.4°, lumbar lordosis (LL) = −60.5°. (**B**) immediate postoperative X-rays, PI = 57.8°, TK = 24.7°, LL = −48.3°; ideal LL = 0.7*57.8 + 0.4*24.7 + 1 = −51.3°, ΔLL = 48.3–51.3 = 3.0°. (**C**) no mechanical complications at 2-year follow-up.

**Figure 5:**
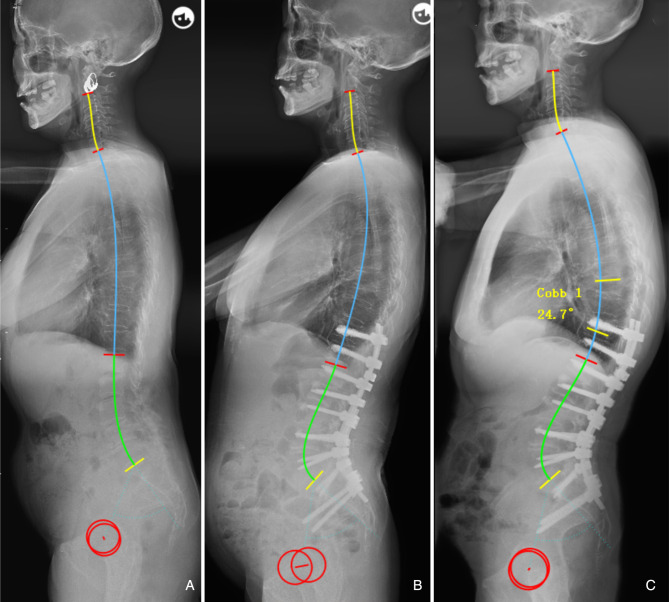
66-year-old female. (**A**) preoperative sagittal standing full spine X-rays, pelvic incidence (PI) = 57.6°, thoracic kyphosis (TK) = 19.9°, lumbar lordosis (LL) = −36.1°. (**B**) immediate postoperative X-rays, pelvic incidence = 56.6°, thoracic kyphosis = 27.1°, lumbar lordosis (LL) = −65.1°; ideal LL = 0.7*56.6 + 0.4*27.1 + 1 = −51.5°, ΔLL = 65.1–51.5 = 13.6°. (**C**) PJK occurred at 6-month follow-up.

**Figure 6:**
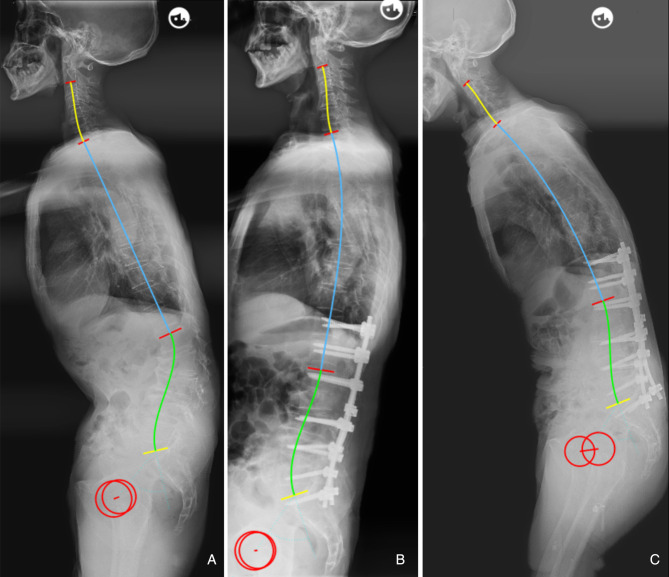
65-year-old male (**A**) preoperative sagittal standing full spine X-rays, pelvic incidence (PI) = 53.6°, thoracic kyphosis (TK) = 2.4°, lumbar lordosis (LL) = 11.5°. (**B**) immediate postoperative X-rays, PI = 53.3°, TK = 15.5°, LL = −26.3°; ideal LL = 0.7*53.3 + 0.4*15.5 + 1 = −44.5°, ΔLL = 26.3–44.5 = 18.2°. (**C**) Distal screw pull-out at 2.5-year follow-up.

**Figure 7:**
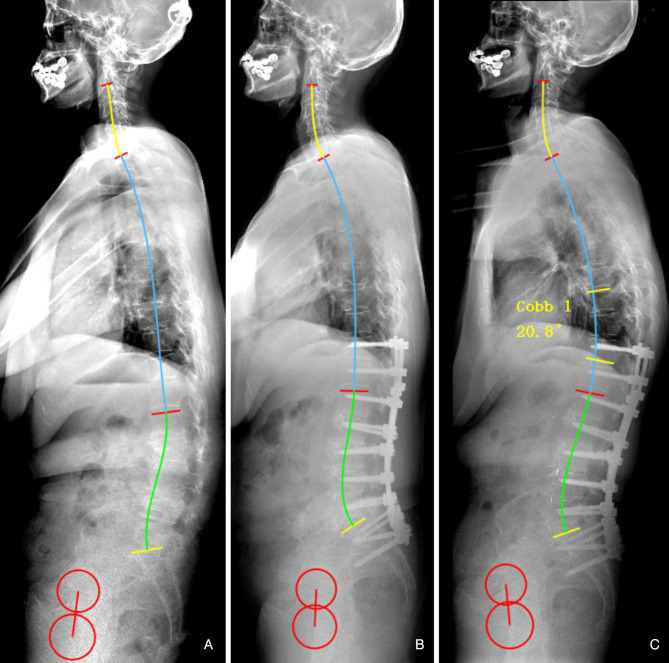
60-year-old female. (**A**) preoperative sagittal standing full spine X-rays, pelvic incidence (PI) = 57.3°, thoracic kyphosis (TK) = 4.4°, lumbar lordosis (LL) = −2.6°. (**B**) immediate postoperative X-rays, pelvic incidence = 57.7°, thoracic kyphosis = 23.8°, lumbar lordosis (LL) = −34.6°; ideal LL = 0.7*57.7 + 0.4*23.8 + 1 = −50.9°, ΔLL = 34.6–50.9 = 16.3°. (**C**) PJK occurred at 2-year follow-up.

Our findings suggested that the occurrence of postoperative mechanical complications can be partly attributed to a mismatch between the lumbar alignment and the morphology of the pelvis and thoracic spine. Besides, the cut-off value of the predictor was developed. ΔLL* < *9.8° is a protective factor, which indicates that the patient’s sagittal lumbar alignment matches with PI and TK, and helps to reduce mechanical complications caused by mismatched spinopelvic parameters (Figure 4). Conversely, when ΔLL is increased, postoperative lumbar alignment dose not match with PI and TK, abnormal mechanical stress would be imposed on the implants, instrument vertebrae, and proximal unfused segments. In othe words, postoperative sagittal alignment should satisfy the formula LL = 0.7*PI + 0.4*TK + 1 under ideal conditions; when postoperative LL is overcorrected and does not match with PI and TK, it will be accompanied by an excessive increase in TK, which may lead to the occurrence of PJK (Figure 5); for patients whose postoperative LL is undercorrected, TK will be correspondingly in a compensatory state, i.e., by reducing the kyphosis to maintain LL matching PI and TK. However, when inadequate LL surpasses the compensative ability of unfused segments, mechanical complications and even sagittal imbalance will occur (Figures 6, 7).

We infer that the formula has the following important aspects. First, it demonstrates that LL should be matched for both PI and TK, and thus can be used by surgeons to understand the mechanisms of lumbar alignment generation. Second, TK is admittedly not completely constant, unlike PI, and may have pathological variation prior to surgery; thus, most important function of this formula is to predict the probability of developing mechanical complications based on the postoperative parameters of the sagittal shape, thereby guiding on follow up and effective intervention. Previous literatures suggested that the optimal PI-LL is not a constant but rather a flexible value determined by individual PI (28, 29). Yilgor et al.(21) believed that the concept of using PI-LL solely as an absolute numeric value with mean thresholds based on population may be misleading, which can aslo be demonstrated by our results. For example, postoperative LL was within a 10° range of the PI in both Figures 4, 5, however, the two patients had different outcomes. Im et al. (30) found that the degree of LL correction relative to PI was not associated with PJK prevalence in elderly patients with ASD, which also suggested that PI-LL may not be a good predictor of mechanical complications. In our study, the novel formula has advantages in predicting mechanical complications because it is established based on individual sagittal parameters of age specific population and takes into account the interaction between TK and LL.

Some limitations of this study must be discussed. First, given the strict inclusion and exclusion criteria applied, the sample size of elderly asymptomatic patients enrolled was relatively small, which might lead to selection bias. Moreover, the results obtained may not necessarily be applicable to other regions and clinical contexts. Second, some demographic and surgical factors that may affect mechanical complications such as bone mineral density, muscle strength, and surgical technique were not analyzed. Third, we evaluated radiographic and instrumentation-related complications as a whole because of their overlapping biomechanical properties. However, the ability of the model to predict each type of mechanical complication was not been explored. Finally, TK is hard to determine without fixing all thoracic vertebrae due to reciprocal changes (RC) of unfused segments. Adverse RC may limit optimal correction (31). Thus, in terms of preoperative design, our formula may be more suitable for patients who require a long instrumented fusion from the proximal thoracic spine to the pelvis to achieve ideal global sagittal realignment. For patients who will undergo fusions from the lower thoracic spine to the sacrum/iliac, surgeons need to recognize the physiological TK under the mobility of the thoracic spine to reconstruct a more reasonable lumbar alignment, which requires further verified in long-term follow-up prospective studies with large sample size.

## Conclusions

Ideal lumbar lordosis should be matched for both PI and TK. The prediction formula of LL based on PI and TK in asymptomatic adults was established, which is expected to help surgeons to comprehend the mechanisms of lumbar alignment generation and predict occurrence of mechanical complications after ASD surgery.

## Data Availability

The raw data supporting the conclusions of this article will be made available by the authors, without undue reservation.
